# Transactions of the Brooklyn Dental Association

**Published:** 1864-09

**Authors:** W. C. Horne


### TRANSACTIONS OF THE BROOKLYN DENTAL ASSOCIATION.

REPORTED BY DR. W. C. HORNE.

June 22d, 1864.

The Society met at the house of Dr. W. H. Atkinson. The greater part of the evening was consumed in the consideration of business matters. Dr. Marvin read a paper entitled, "The Dentist's Duty to his Patients," which was well received and requested for immediate publication. At a late hour the subject of "Dental Charges," continued from the last meeting, was taken up.

Dr. C. P. Fitch said the theme was perplexing and hackneyed; there can be no standard of prices for the reason that every one views the subject from his own stand-point. The Dentist should be honest and faithful in considering the quality of his service and the ability of his patient. The vexed question is, what is extortionary? It is extortionate to ask high prices for poor work. Every man should set his own minimum and maximum. He (the speaker) arranged his prices according to the number of grains of gold used.

Dr. W. B. Hurd. This *is* a vexed question, but none the less an important one. Every man should be remunerated for his labor. The patient should not rob his Dentist by withholding his due, neither should the Dentist take advantage of his patient by demanding, after the work is done, more than he has any right to. There is such a thing as a proper maximum price, a tooth *may* last fifty years, owing to its being well filled, but we should not value our work by such a conjecture. It is sound policy to charge reasonable prices: it is much better that a man should earn his living by six or eight hours honest labor, than by the effort of a single hour at an extortionate price.

Dr. C. P. Fitch. A man who has the ability should pay such prices as that every necessary outlay of time and skill may be bestowed by his Dentist.

Dr. Atkinson had often asked himself "Can I make a rule by which to govern my charges?" He had been unable to do so; and he had not heard any of the gentlemen who had spoken name a standard. No man who is worthy of the name, will, for his own case, set the comfort of his fellow-men at defiance. Every man decides all questions by his highest standard of good; even though he be the veriest infidel this is true. The matter of price takes care of itself in the commonest things; why not in the most uncommon? and how can a man determine what his price shall be before he can take into consideration the circumstances which shall determine it? Before entering upon any operation it was his custom to ask himself—Am I prepared to do this work so that every energy of my mind and body may be brought to bear upon it?" and when it is thus done he asks himself "What ought to be my remuneration?" Professional men ought to be honorable enough to be trusted to answer this question for themselves. He would say that he *had* operated gratuitously, and felt more richly paid by a few simple words of thanks from a young girl than when a golden fee had been counted out to him of $100 or $200.

When our religion enters our work and we say like Peter " Such as I have give I unto thee," then we are safe operators. He was sorry there was no more religion in every day work; we might all be a loving band of brothers and bring the other side of Jordan here. He had in the early days of his practice put in fillings at fifty cents, some of which did service to the present day; then he had not learned to put a proper estimate upon his services. No man can tell what an operation is worth unless he has witnessed all its stages; for the value of the whole depends on the perfection of each step in the process.

The Society then adjourned.

